# Localization of organic anion transporting polypeptide (Oatp) 1a4 and Oatp1c1 at the rat blood-retinal barrier

**DOI:** 10.1186/2045-8118-10-29

**Published:** 2013-10-02

**Authors:** Shin-ichi Akanuma, Shiro Hirose, Masanori Tachikawa, Ken-ichi Hosoya

**Affiliations:** 1 Department of Pharmaceutics, Graduate School of Medicine and Pharmaceutical Sciences, University of Toyama, 2630 Sugitani, Toyama 939-0364, Japan; 2 Division of Membrane Transport and Drug Targeting, Graduate School of Pharmaceutical Sciences, Tohoku University, Aoba, Aramaki, Aoba-ku, Sendai, Miyagi 980-8578, Japan

**Keywords:** Inner blood-retinal barrier, Outer blood-retinal barrier, Blood–brain barrier, Organic anion transporting polypeptide, Oatp1a4, Oatp1c1

## Abstract

**Background:**

Organic anion transporting polypeptide (Oatp) transporters at the blood–brain barrier (BBB) and the blood-retinal barrier (BRB), which consists of retinal capillary endothelial cells and retinal pigment epithelial cells, are major determinants of the control of anionic drugs into the brain and retina. Although Oatp1a4 (Slco1a4) and Oatp1c1 (Slco1c1) are known to be expressed in the abluminal and luminal membrane of the rat BBB and Oatp1a4 is known to be expressed at the BRB, the expression and localization of Oatp1c1 at the BRB and subcellular localization of Oatp1a4 at the BRB have received little attention. Therefore, the purpose of present study was to determine the cellular and subcellular localization of Oatp1a4 and 1c1 at the BRB.

**Methods:**

We used guinea pig polyclonal antibodies to Oatp1a4 and 1c1 for immunoblot and immunohistochemical analysis to determine their cellular and subcellular distributions in the rat retina. We compared these distributions with those of the glucose transporter 1 (GLUT1/Slc2a1). Whole brain, brain capillary fractions and kidney were used as control.

**Results:**

Oatp1a4 and 1c1 immunoreactivities were detected in the rat retinal capillaries and co-localized with GLUT1, suggesting that both proteins are located on the abluminal and luminal membrane of the retinal capillary endothelial cells. Oatp1a4 and 1c1 immunoreactivities were preferentially detected on the apical and basolateral membrane of rat retinal pigment epithelial cells, respectively, suggesting that Oatp1a4 and 1c1 are localized on the apical membrane and the basolateral membrane of the retinal pigment epithelial cells, respectively.

**Conclusion:**

Oatp1a4 and 1c1 are present at the BRB and contribute to the transcellular transport of amphipathic organic anions across the BRB.

## Background

In the retina, the blood-retinal barrier (BRB), which is formed by complex tight junctions of retinal capillary endothelial cells (inner BRB) and retinal pigment epithelial cells (RPE; outer BRB), control the anatomical, biochemical, and transport mechanisms that regulate the access of molecules in the circulating blood to the neural retina [1,2]. Similarly, the brain has two barriers: the blood–brain barrier (BBB) and the blood-cerebrospinal fluid barrier (BCSFB) which consist of brain capillary endothelial cells and choroid plexus epithelial cells, respectively [3]. The tight junctions between cells of the BRB and RPE at these boundaries create physical barriers to diffusion of substances from the circulating blood to the retina. However, the BRB is not an impermeable barrier since essential nutrients are efficiently transferred to the retina from the circulating blood, and endobiotics and xenobiotics are selectively removed from the retina across the BRB [1,4]. The inner and outer BRB express a variety of unique transporters which play a pivotal role in the influx transport of essential molecules and the efflux transport of hormones, neurotransmitter metabolites, and drugs.

*In vivo* studies using microdialysis, have shown that the elimination of amphipathic organic anions, such as estradiol 17-β glucuronide (E17βG) and dehydroepiandrosterone sulfate (DHEAS), from the vitreous humor/retina involves saturable mechanisms [1,5], suggesting that organic anion transporting polypeptides (Oatps) are involved in the uptake of E17βG and DHEAS at the BRB. Mammalian Oatps exhibit wide tissue expression with emphasis on their expression in barrier cells [6]. Our previous study revealed that Oatp1a4 (Oatp2/Slco1a4) and Oatp1c1 (Oatp14/Slco1c1) mRNAs are highly expressed in rat retinal capillary endothelial cells compared with other retinal cells [7]. Oatp1a4 and 1c1 exhibit broad substrate specificity for amphipathic compounds and Oatp1a4 has a high affinity for the cardiac glycoside, digoxin [8]. Immunohistochemical staining of rat brain has shown that Oatp1a4 and 1c1 are localized in both the abluminal and luminal membrane of the BBB [9,10]. These results and kinetic analyses suggest that Oatp1a4 and 1c1 mediate uptake from both brain and blood. At the BCSFB, Oatp1a4 and 1c1 have been reported to be localized at the basolateral membrane of choroid plexus epithelial cells [9,10]. In the retina, although Oatp1a4 is known to be localized at the apical membrane of the RPE and is expressed in the retinal capillary endothelial cells [7,11,12], there is little information available about the expression and localization of Oatp1c1 at the BRB and the subcellular localization of Oatp1a4 at the inner BRB.

The purpose of this study was to determine the cellular and subcellular localization of Oatp1a4 and 1c1 at the BRB. We have produced specific antibodies to Oatp1a4 and 1c1, investigated the expression of Oatp1a4 and 1c1, and compared their distribution with that of glucose transporter 1 (GLUT1/Slc2a1).

## Methods

### Animals

Male Wistar rats (100–200 g) and female Hartley guinea pigs (300–400 g) were purchased from Japan SLC (Hamamatsu, Japan). The investigations using animals described in this report conformed to the provisions of the Animal Care Committee, University of Toyama and the Association for Research in Vision and Ophthalmology Statement on the Use of Animals in Ophthalmic and Vision Research.

### Antibody preparation

Polyclonal antibodies to Oatp1a4 and 1c1 were raised against amino acid residues 625–661 of rat Oatp1a4 (GenBank accession number: NP_571981) and 1–35 of rat Oatp1c1 (GenBank accession number: NP_445893.1). The specificity of these amino acid sequences was confirmed by NCBI protein-protein BLAST search against the dataset of all non-redundant protein sequences. These polypeptides were expressed as glutathione S-transferase (GST) fusion proteins using the pGEX4T-2 plasmid vector (GE Healthcare, Chalfont St. Giles, UK). The fusion protein was purified with glutathione-Sepharose 4B (GE Healthcare), emulsified with Freund’s complete adjuvant (Difco, Detroit, MI, USA), and injected subcutaneously into female Hartley guinea pigs at intervals of 2 weeks. Two weeks after the sixth injection, affinity-purified antibodies were prepared, first using protein G-Sepharose (GE Healthcare) and then using antigen peptides coupled to cyanogens bromide-activated Sepharose 4B (GE Healthcare). For the preparation of affinity media, polypeptides free of GST were obtained by elution of the cleaved polypeptide after in-column thrombin digestion of fusion proteins bound to glutathione-Sepharose 4B. Guinea pig polyclonal anti-Oatp1a4 antibody was prepared in previous study [13].

### Immunoblot analysis

Under pentobarbital anesthesia (50 mg/kg bodyweight, i.p.), rats were transcardially perfused with phosphate-buffered saline (PBS). Rat brain capillary fraction was prepared as described previously with minor modification [14]. In brief, cerebrum was excised, cut into 2 mm pieces, and homogenized in PBS using a Potter-Elvehjem homogenizer. The homogenate was added to the same volume of 32% dextran solution, and the mixture was centrifuged (4,500 g, 10 min, 4°C). The resulting pellets were washed with PBS and suspended in Tris/sucrose buffer (250 mM sucrose, 10 mM Tris–HCl, and 1 mM EGTA, pH 7.4).

The retina, brain, brain capillary fraction, and kidney were homogenized using the nitrogen cavitation technique (800 psi, 30 min, 4°C) in Tris/sucrose buffer containing protease inhibitor cocktail (Sigma, St. Louis, MO, USA), and homogenized samples were centrifuged at 10,000 g for 15 min. The supernatants were then centrifuged at 100,000 g for 1 h, and a crude membrane fraction was obtained from the pellets. The protein concentration in each sample was determined using a DC protein assay kit (Bio-Rad, Hercules, CA, USA). Crude membrane protein (5 μg/lane or 20 μg/lane) was separated on a sodium dodecyl sulfate (SDS)-polyacrylamide gel and subsequently electrotransferred to a polyvinylidene difluoride membrane. Following incubation with Tris-buffered saline (TBS; 25 mM Tris–HCl and 125 mM NaCl, pH 7.4) containing 0.1% Tween 20 and 5% skimmed milk for 12–16 h at 4°C, the membranes were incubated with guinea pig polyclonal anti-Oatp1a4 antibody (0.1 μg/mL; [13]) or guinea pig polyclonal anti-Oatp1c1 antibody (0.1 μg/mL) for 3 h at 15–25°C. Antigen absorption was performed by incubating guinea pig anti-Oatp1a4 antibody or anti-Oatp1c1 antibody with respective GST-fused antigen (10 μg/mL) for 1 h at 4°C. The membranes were subsequently incubated with horseradish peroxidase-conjugated anti-guinea pig antibody. The bands were then visualized with an enhanced chemiluminescence kit (ECL Prime Western Blotting Detection System; GE healthcare).

### Immunohistochemical analysis

Under pentobarbital anesthesia (100 mg/kg body weight, i.p.), rats were perfused transcardially with 4% formaldehyde in 0.1 M phosphate buffer (pH 7.4). Then their eyeballs or brains were isolated and immersed in 30% sucrose/phosphate buffer. Frozen sections (15 μm in thickness) were cut from the frozen eye or brain using a cryostat (CM1900; Leica, Heidelberg, Germany), mounted on silanized glass slides (DAKO, Carpinteria, CA, USA), and air-dried. Following incubation with 10% goat serum (Nichirei, Tokyo, Japan) for 1 h at room temperature, sections were incubated with guinea pig polyclonal anti-Oatp1a4 antibody (1 μg/mL [13]) or guinea pig polyclonal anti-Oatp1c1 antibody (2 μg/mL) and rabbit polyclonal anti-GLUT1 antibody (0.5 μg/mL [15]) for 12 h at 4°C. Sections were subsequently incubated with Alexa Fluor 488-conjugated (1:200; Life Technologies, Carlsbad, CA) and Cy3-conjugated secondary antibodies (1:200; Merck Millipore, Billerica, MA) for 2 h at room temperature. Nuclei were stained by incubating with 4 μM 4',6-diamidino-2-phenylindole in PBS(-) for 5 min at room temperature.

Images were captured using a confocal laser microscope (TCS-SP5; Leica) equipped with a blue diode/argon/green diode laser system. To avoid bleed-through into adjacent detection channels, 4',6-diamidino-2-phenylindole, Alexa Fluor 488, and Cy3 were excited sequentially using the 405, 488, and 561 nm excitation laser wavelengths, respectively. Emissions were collected using the spectral detection system, configured with a galvanometer diffraction grating in combination with a variable slit for high-resolution wavelength separation. Images were acquired using an appropriate pinhole to obtain 1 Airy unit. All images were captured using a confocal software (LAS AF, Leica), digitized at 8-bit resolution into an array of 1024 × 1024 pixels.

### Data analysis

Using the confocal software (LAS AF), the line scanning of images was carried out, and the co-localization studies were performed by the overlap coefficient, proposed by Manders *et al.*[16].

## Results

### Localization of Oatp1a4 in the rat retina

**Figure 1:**
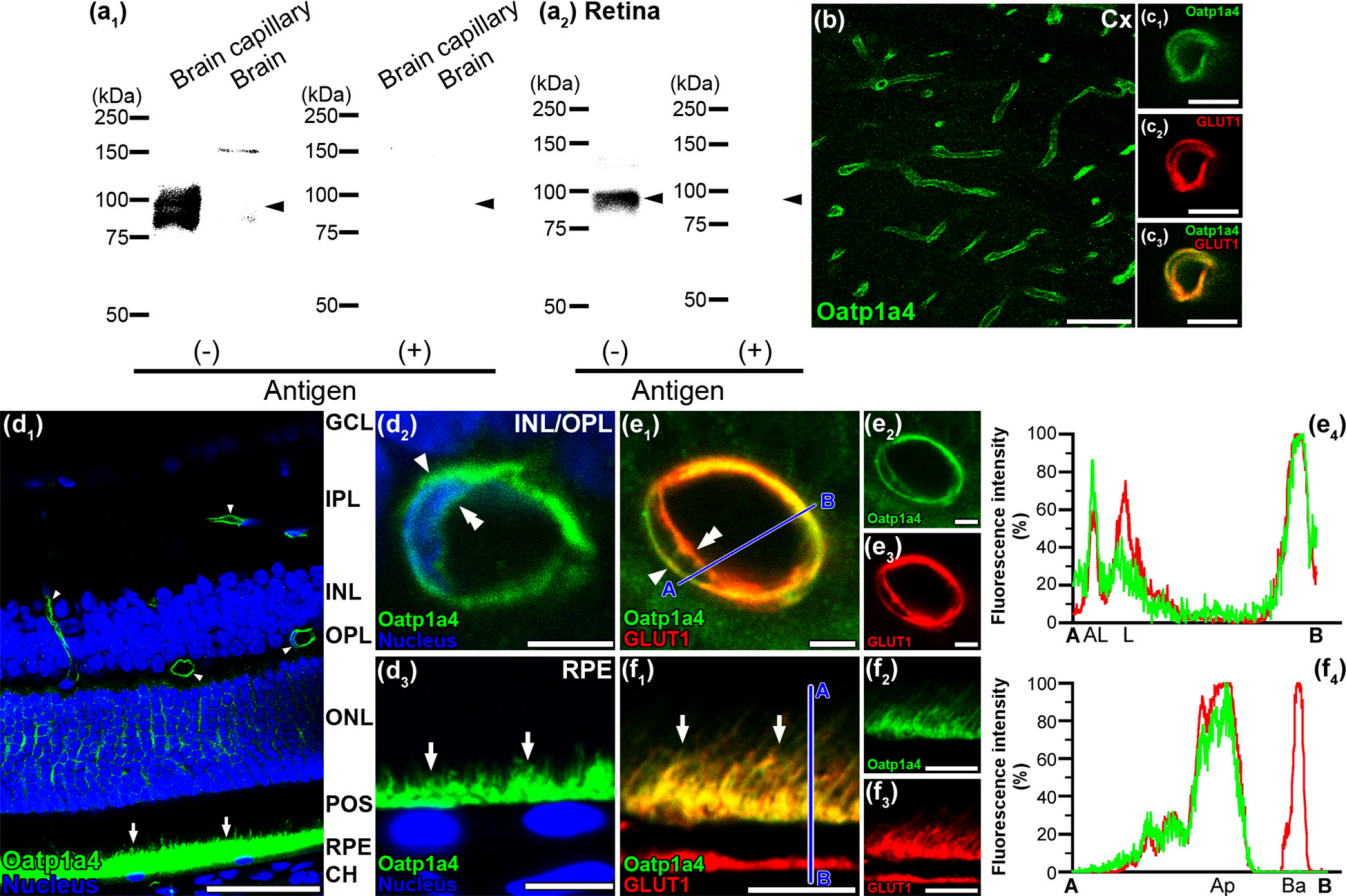
**Expression and localization of Oatp1a4 in rat brain/retinal capillary endothelial cells and RPE. (a)** Immunoblotting with Oatp1a4 antibodies using proteins (5 μg/lane) prepared from rat brain capillary fraction, brain **(a**_**1**_**)**, and retina **(a**_**2**_**)** in the absence [antigen(-)] or presence [antigen(+)] of Oatp1a4 antigens. Arrow heads indicates the expected position of the band. **(b)** Oatp1a4 immunofluorescence in the rat cerebral cortex (Cx). **(c)** Oatp1a4 (green, **c**_**1**_ and **c**_**3**_) overlapped with GLUT1 (red, **c**_**2**_ and **c**_**3**_) in brain capillaries. **(d)** Oatp1a4 (green) in the retinal capillaries (arrow heads and double arrow heads) and the RPE (arrows) of rat retina. Oatp1a4 was observed around the nucleus (blue) of retinal capillaries (arrow head and double arrow head, **d**_**2**_) and above the nucleus of the RPE (arrows, **d**_**3**_). **(e)** Oatp1a4 (green, **e**_**1**_ and **e**_**2**_) overlapped with GLUT1 (red, **e**_**1**_ and **e**_**3**_) on the abluminal (arrow head) and luminal membrane (double arrow head) of retinal capillaries. The blue line between A and B indicates the scanned line **(e**_**1**_**)**, and the relative fluorescence intensity is plotted in green for Oatp1a4 and red for GLUT1 **(e**_**4**_**)**, which is expressed in the abluminal (AL) and luminal (L) membrane of the retinal capillaries. **(f)** Oatp1a4 (green, **f**_**1**_ and **f**_**2**_) overlapped with GLUT1 (red, **f**_**1**_ and **f**_**3**_) on the apical membrane (arrows) of rat RPE. The blue line between A and B indicates the scanned line **(f**_**1**_**)**, and the relative fluorescence intensity is plotted in green for Oatp1a4 and red for GLUT1 **(f**_**4**_**)**, which is expressed in the apical (Ap) and basolateral (Ba) membrane of the RPE. GCL, ganglion cell layer; IPL, inner plexiform layer; INL, inner nuclear layer; OPL, outer plexiform layer; ONL, outer nuclear layer; POS, photoreceptor outer segments; CH, choroid. Scale bar: 50 μm **(b**, **d**_**1**_**)**, 5 μm **(c**, **d**_**2**_, **e)**, 10 μm **(d**_**3**_, **f)**.

A broad band at about 92 kDa was detected with anti-Oatp1a4 antibody in the crude membrane fraction from adult rat brain capillary fraction, whereas no band between 75 and 100 kDa was detected in rat brain (Figure 1a_1_). In addition, a single band at about 92 kDa was detected with anti-Oatp1a4 antibody in the crude membrane fractions from adult rat retina (Figure 1a_2_). These bands were absent after pre-absorbing anti-Oatp1a4 antibody with the antigen peptide (Figure 1a_1_ and 1a_2_). High Oatp1a4 immunoreactivities were detected in the brain capillaries (Figure 1b). Double immunostaining with GLUT1 (red, Figure 1c_2_), which is known to be expressed in both the abluminal and luminal membrane of brain capillaries, showed that Oatp1a4 (green, Figure 1c_1_) overlapped on the abluminal and luminal membrane of the capillaries (yellow, Figure 1c_3_). These features indicate that Oatp1a4 is expressed in both the abluminal and luminal membrane of rat brain capillaries. The localization of Oatp1a4 at the BBB is identical to that described in a previous report [9].

In the retina, immunostaining of Oatp1a4 (green, Figure 1d_1_) was observed along the retinal capillaries in the inner plexiform layer, inner nuclear layer, outer plexiform layer (arrow head) and in the RPE (arrow). The immunoreactivities derived from Oatp1a4 were detected around the nucleus in the retinal capillary endothelial cells (Figure 1d_2_) and above the nucleus in the RPE (Figure 1d_3_). Moreover, double immunostaining with GLUT1 (red, Figure 1e_3_), which is known to be expressed in both the abluminal and luminal membrane of retinal capillaries [17], showed that Oatp1a4 (green, Figure 1e_2_) overlapped on the abluminal (arrow head) and luminal membrane of retinal capillaries (double arrow head, yellow, Figure 1e_1_; overlap coefficient = 0.812). These features indicate that Oatp1a4 is expressed in both the abluminal and luminal membrane of rat retinal capillaries. To assess the distribution of Oatp1a4 in the retinal capillaries, line scanning was performed to measure the fluorescence intensity immunostained for Oatp1a4 (green, Figure 1e_4_) and GLUT1 (red, Figure 1e_4_). The relative fluorescence intensity for Oatp1a4 was strongly observed at the abluminal membrane of the retinal capillaries compared with the luminal membrane, indicating that Oatp1a4 is preferentially localized on the abluminal membrane of the retinal capillaries. In rat RPE, Oatp1a4 immunoreactivities were observed above the nucleus of the RPE (arrow, green, Figure 1d_3_ and 1f_2_), and GLUT1, which is known to be expressed in both the apical and basolateral membranes of RPE [17] (red, Figure 1f_3_), and Oatp1a4 overlapped on the apical membrane of RPE (arrow, yellow, Figure 1f_1_ and 1f_4_) but was below the detection threshold on the basolateral membrane (Figure 1f_4_). The overlap coefficient of the immunoreactivities for Oatp1a4 and GLUT1 in Figure 1f_1_ was obtained to be 0.846. Consequently, these results indicate that Oatp1a4 is dominantly expressed in the apical membrane of rat RPE.

### Localization of Oatp1c1 in the rat retina

**Figure 2:**
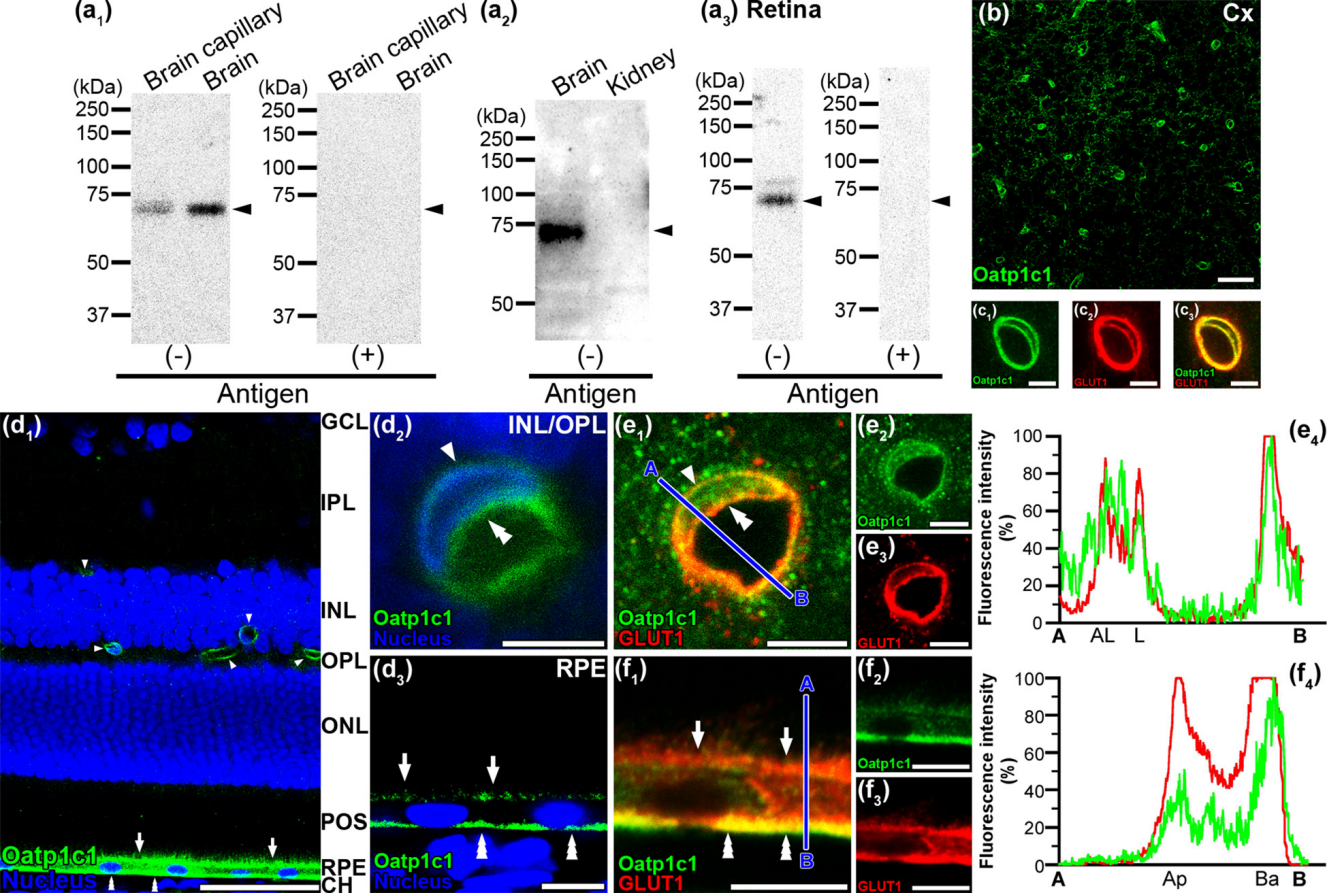
**Expression and localization of Oatp1c1 in rat brain/retinal capillary endothelial cells and RPE. (a)** Immunoblotting with Oatp1c1 antibodies using proteins (20 μg/lane) prepared from rat brain capillary fraction **(a**_**1**_**)**, brain (**a**_**1**_ and **a**_**2**_), retina **(a**_**3**_**)**, and kidney (**a**_**2**_, negative control) in the absence [antigen(-)] or presence [antigen(+)] of Oatp1c1 antigens. Arrow heads indicate the expected position of the band. **(b)** Oatp1c1 immunofluorescence in the rat cerebral cortex (Cx). **(c)** Oatp1c1 (green, **c**_**1**_ and **c**_**3**_) overlapped with GLUT1 (red, **c**_**2**_ and **c**_**3**_) in brain capillaries. **(d)** Oatp1c1 (green) in the retinal capillaries (arrow heads and double arrow heads) and the RPE (arrows and triple arrow heads) of rat retina. Oatp1c1 was observed around the nucleus (blue) of retinal capillaries (arrow head and double arrow head, **d**_**2**_) and around the nucleus of the RPE (arrows and triple arrow heads, **d**_**3**_). **(e)** Oatp1c1 (green, **e**_**1**_ and **e**_**2**_) overlapped with GLUT1 (red, **e**_**1**_ and **e**_**3**_) on the abluminal (an arrow head) and luminal membrane (a double arrow head) of retinal capillaries. The blue line between A and B indicates the scanned line **(e**_**1**_**)**, and the relative fluorescence intensity is plotted in green for Oatp1c1 and red for GLUT1 **(e**_**4**_**)**, which is expressed in the abluminal (AL) and luminal (L) membrane of the retinal capillaries. **(f)** Oatp1c1 (green, **f**_**1**_ and **f**_**2**_) overlapped with GLUT1 (red, **f**_**1**_ and **f**_**3**_) on the apical (arrows) and basolateral membrane (triple arrow heads) of rat RPE. The blue line between A and B indicates the scanned line **(f**_**1**_**)**, and the relative fluorescence intensity is plotted in green for Oatp1c1 and red for GLUT1 **(f**_**4**_**)**, which is expressed in the apical (Ap) and basolateral (Ba) membrane of the RPE. Scale bar: 50 μm **(b**, **d**_**1**_**)**, 5 μm **(c**, **d**_**2**_, **e)**, 10 μm **(d**_**3**_, **f)**.

Using immunoblotting with the crude membrane fraction from rat brain capillary fraction and brain, anti-Oatp1c1 antibody was recognized as a single band at 73 kDa, but not with kidney (Figure 2a_1_ and 2a_2_). The size of band detected was consistent with previous results [18], indicating the high specificity of the antibody for rat Oatp1c1. A single band at about 73 kDa with anti-Oatp1c1 antibody was also detected in the retina (Figure 2a_3_). These bands were absent after pre-absorbing anti-Oatp1c1 antibody with the antigen peptide (Figure 2a_1_ and 2a_3_). Oatp1c1 immunoreactivities were detected in brain capillaries (Figure 2b). Double immunostaining with GLUT1 (red, Figure 2c_2_), which is known to be expressed in both the abluminal and luminal membrane of brain capillaries, showed that Oatp1c1 (green, Figure 2c_1_) overlapped on the abluminal and luminal membrane of capillaries (yellow, Figure 2c_3_). These features indicate that Oatp1c1 is expressed in both the abluminal and luminal membrane of rat brain capillaries and the localization of Oatp1c1 at the BBB is identical to that described in a previous report [10].

In the retina, immunostaining of Oatp1c1 (green, Figure 2d_1_) was observed along the retinal capillaries in the inner plexiform layer, inner nuclear layer, inner and outer plexiform layer (arrow head), and in the RPE (arrow and triple arrow head). The immunoreactivities derived from Oatp1c1 were detected around the nucleus in the retinal capillary endothelial cells (Figure 2d_2_) and the RPE (Figure 2d_3_). Double immunostaining with GLUT1 (red, Figure 2e_3_), which is known to be expressed in both the abluminal and luminal membrane of retinal capillaries [17], showed that Oatp1c1 (green, Figure 2e_2_) overlapped on the abluminal (arrow head) and luminal membrane of retinal capillaries (double arrow head, yellow, Figure 2e_1_, overlap coefficient = 0.789). To evaluate the intracellular distribution of Oatp1c1 in the retinal capillary endothelial cells, line scanning was performed to measure the fluorescence intensity immunostained for Oatp1c1 (green, Figure 2e_4_) and GLUT1 (red, Figure 2e_4_). The relative fluorescence intensity for Oatp1c1 showed peaks not only inside the cytoplasm but also at the luminal and abluminal membrane of the retinal capillary endothelial cells. Thus, it is indicated that Oatp1c1 is expressed in both the abluminal and luminal membrane of the retinal capillaries. Oatp1c1 immunoreactivity (green, Figure 2d_3_ and 2f_1_) was observed along both the retinal (arrow) and choroid sides (triple arrow head) of the RPE and GLUT1, which is known to be expressed in both the apical and basolateral membrane of the RPE [17] (red, Figure 2f_3_), showed that Oatp1c1 mainly overlapped on the basolateral membrane of the RPE (triple arrow head, yellow, Figure 2f_1_; overlap coefficient = 0.795). The relative fluorescence intensity for Oatp1c1 at the basolateral membrane of the RPE is greater than that at the apical membrane of the RPE (Figure 2f_4_). These results indicate that Oatp1c1 is preferentially expressed in the basolateral membrane of rat RPE.

## Discussion

The present study demonstrates that retinal capillary endothelial cells and retinal pigment epithelial cells express proteins for Oatp1a4 and 1c1 (Figures 1 and 2). The presence of these transporters at the inner and outer BRB leads us to propose that Oatp1a4 and 1c1 are able to transport amphipathic organic anions in the retina.

We further demonstrated the immunoreactivities of Oatp1a4 and 1c1 on the inner and outer sides of the endothelium nuclei along with GLUT1 which is a marker for both the abluminal and luminal membrane of retinal capillaries [17] and showed that Oatp1a4 and 1c1 are located on both the abluminal and luminal membrane of retinal capillary endothelial cells (Figures 1e_1_ and 2e_1_). These expressional patterns are similar to the finding that Oatp1a4 and 1c1 are localized on both the abluminal and luminal membrane of brain capillary endothelial cells (Figures 1c_3_ and 2c_3_) [9,10]. Oatp1a4 and 1c1 are preferentially located on the apical and basolateral membrane, respectively, of rat RPE. The localization of Oatp1a4 at the RPE is consisted with a previous report [12].

Oatp1a4 and 1c1 mediate the cellular uptake of amphipathic organic anions from the brain side and the blood side of the BBB [10,19]. In the retina, when [^3^H]E17βG was injected into the vitreous humor, its subsequent elimination from the vitreous humor/retina across the BRB was carrier-mediated, and the elimination was completely inhibited by the presence of probenecid, while digoxin had a much weaker effect [5]. Our immunohistochemical studies indicate that Oatp1a4 is preferentially expressed at the retinal side of the inner and outer BRB (Figure 1e and 1f). Although Oatp1a4 on the retinal side of the inner and outer BRB is involved in the uptake of E17βG from the retina, the partial inhibition by digoxin suggested that an additional uptake transporter, such as Oatp1c1, on the retinal side of the inner BRB is involved in the uptake of E17βG from the retina. When L-[^125^I]-thyroxin, ([^125^I]T_4_) was injected into the carotid artery, the subsequent uptake of [^125^I]T_4_ into the brain was inhibited in the presence of 50 μM T_4_, but not into the retina [20], suggesting that Oatp1c1 on the luminal side of the BBB, but not the BRB, is involved in the uptake of T_4_ from the circulating blood. The sensitivity of Oatp1c1 on the luminal and basolateral membrane of the inner and outer BRB is less than that of the BBB. Further studies are needed to determine concentration-dependent inhibition of the uptake of [^125^I]T_4_ by the retina or brain.

## Conclusion

Oatp1a4 and 1c1 are respectively localized on both the abluminal and luminal membrane of rat retinal capillary endothelial cells, and on the apical and basolateral membrane of the RPE. Our results support the hypothesis that Oatp1a4 and 1c1 contribute to the transcellular transport of amphipathic organic anions across the BRB in both the blood-to-retina and retina-to-blood directions.

## Abbreviations

BBB: Blood–brain barrier; BCSFB: Blood-cerebrospinal fluid barrier; BRB: Blood-retinal barrier; CH: Choroid; Cx: Cerebral cortex; DHEAS: Dehydroepiandrosterone sulfate; E17βG: Estradiol 17-β glucuronide; GCL: Ganglion cell layer; GLUT1: Glucose transporter 1; GST: Glutathione S-transferase; INL: Inner nuclear layer; IPL: Inner plexiform layer; Oatp: Organic anion transporting polypeptide; ONL: Outer nuclear layer; OPL: Outer plexiform layer; PBS: Phosphate-buffered saline; POS: Photoreceptor outer segments; RPE: Retinal pigment epithelial cells; SDS: Sodium dodecyl sulfate; T4: L-thyroxin; TBS: Tris-buffered saline.

## Competing interests

The authors declare that they have no competing interests.

## Authors’ contributions

SA carried out the animal studies, data analysis, and the manuscript preparation. SH carried out the antibody’s preparation and animal studies. MT carried out the antibody preparation and helped to draft the manuscript. KH supervised the study design and manuscript preparation. All authors read and approved the final manuscript.
